# Rare case of dual lesion: intraosseous microcystic meningioma – case report and review of literature

**DOI:** 10.1186/s13256-026-06354-8

**Published:** 2026-08-06

**Authors:** Haijuan Teng, Xuejie Li, Xiaoxia Zhang

**Affiliations:** 1 Department of Radiology, Zhejiang University School of Medicine First Affiliated Hospital, 79 Qingchun Road, Hangzhou, 310003 Zhejiang China; 2 Department of Pathology, Zhejiang University School of Medicine First Affiliated Hospital, 79 Qingchun Road, Hangzhou, 310003 Zhejiang China

**Keywords:** Intraosseous meningioma, Microcystic variant, Skull lesion, Case report, MRI

## Abstract

**Introduction/background:**

Intraosseous microcystic meningioma is an exceptionally rare clinical entity, representing a diagnostic challenge due to its atypical osteolytic presentation and specific histological features. This case is unique because it occurs in a 43-year-old patient—younger than the reported median age—and highlights specific MRI "shine-through" effects that can assist in preoperative differentiation from aggressive bone malignancies.

**Case presentation:**

A 43-year-old woman presented with a six-month history of intermittent parietal pain. Imaging revealed a 1.9 × 0.9 cm osteolytic defect in the right frontal bone with internal septations. MRI showed T2-hyperintensity and heterogeneous enhancement, but no restricted diffusion on ADC maps. The patient underwent gross total resection with dural repair and titanium mesh reconstruction. Histopathology confirmed a WHO Grade I microcystic meningioma, positive for EMA, SSTR2, and D2-40.

**Conclusion:**

The main "take-away" is that intraosseous microcystic meningioma should be considered in the differential diagnosis of solitary osteolytic skull lesions. Multi-parametric MRI, specifically the absence of restricted diffusion despite high DWI signal (T2 shine-through), is a key indicator of this benign, fluid-rich subtype.

## Introduction

Meningiomas are among the most prevalent primary intracranial neoplasms, accounting for approximately 13% to 26% of all primary brain tumors [1, 2]. However, primary extradural meningiomas remain an outlier in clinical practice. Specifically, those arising within the skull—termed primary intraosseous meningiomas (PIMs)—are exceedingly rare [3–6]. According to the classification by Lang et al., these are divided into osteoblastic and osteolytic subtypes [7]. The latter, characterized by bone destruction, often presents a diagnostic quandary as it mimics more aggressive lytic lesions.

The microcystic variant is a pathologically distinct subtype, representing roughly 1.6% of all meningioma cases [8–10]. Histologically, it is defined by a loose, vacuolated architecture with intercellular microcysts, which often manifests as atypical signal patterns on MRI [11]. The intersection of an intraosseous location and microcystic histology is a clinical rarity, with only a handful of cases documented in current literature [7, 11–14]. Herein, we report a new case of intraosseous microcystic meningioma to further characterize this rare entity. The clinical presentation, imaging features, and histopathological findings are described in detail, with the aim of increasing awareness of this unusual tumor and assisting in the differential diagnosis of osteolytic skull lesions.

## Case presentation

A 43-year-old woman presented with a several-month history of intermittent dull pain in the parietal region. The discomfort had begun approximately six months earlier without any identifiable trigger, was mild in intensity, and tended to resolve spontaneously. She had not sought medical attention for the symptom prior to this evaluation.

Her family history was unremarkable, with no known history of meningioma or other brain tumors among her parents or siblings. She denied smoking, alcohol abuse, or illicit drug use. She lives with her husband and works as an office clerk. On examination, she was alert and oriented, with a supple neck. Neurological assessment revealed pupils that were equal and reactive to light, normal muscle strength in all extremities, and no focal deficits. The timeline of this case is presented in Table 1.

**Table 1:**
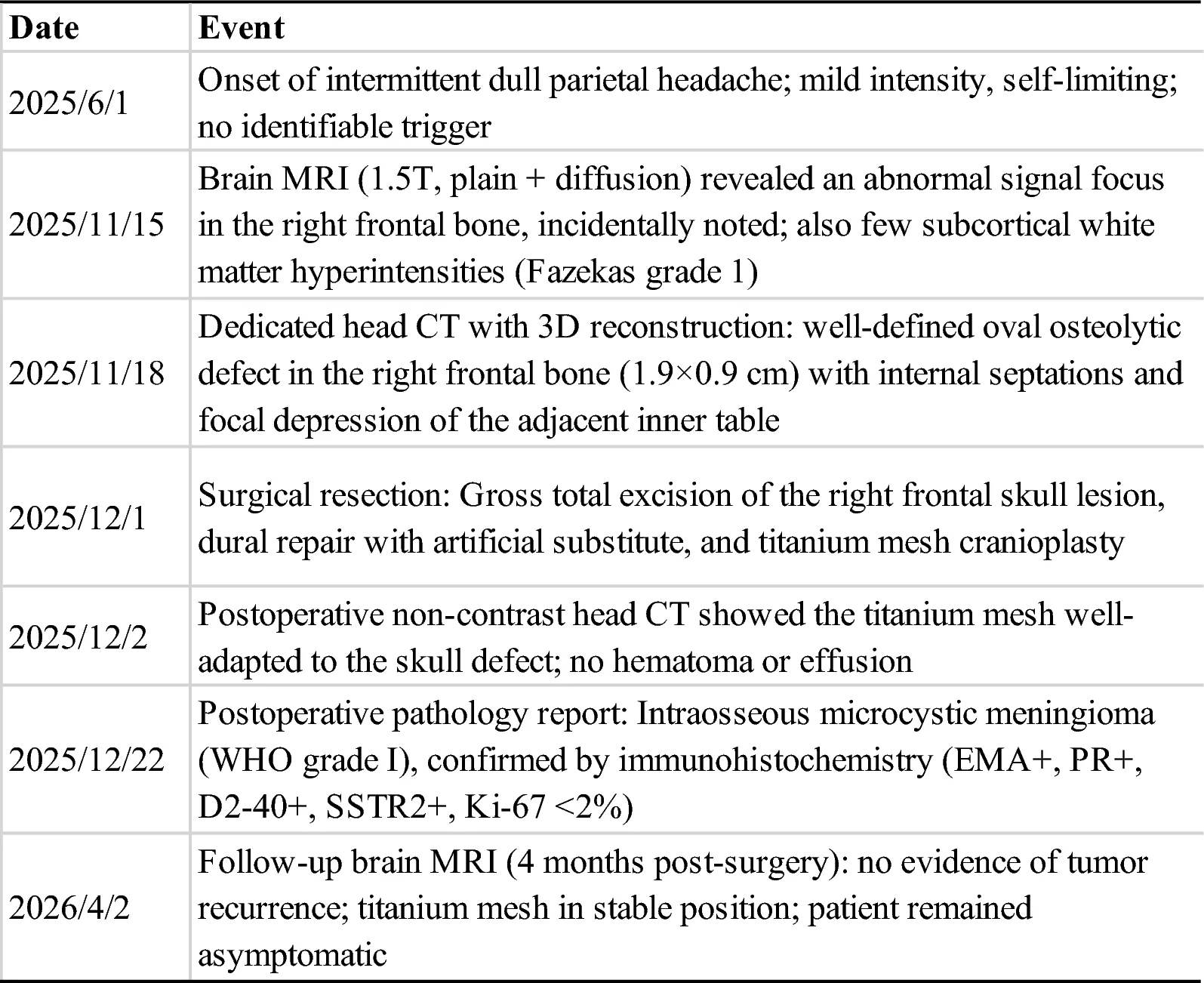
Timeline

### Imaging findings

**Fig. 1:**
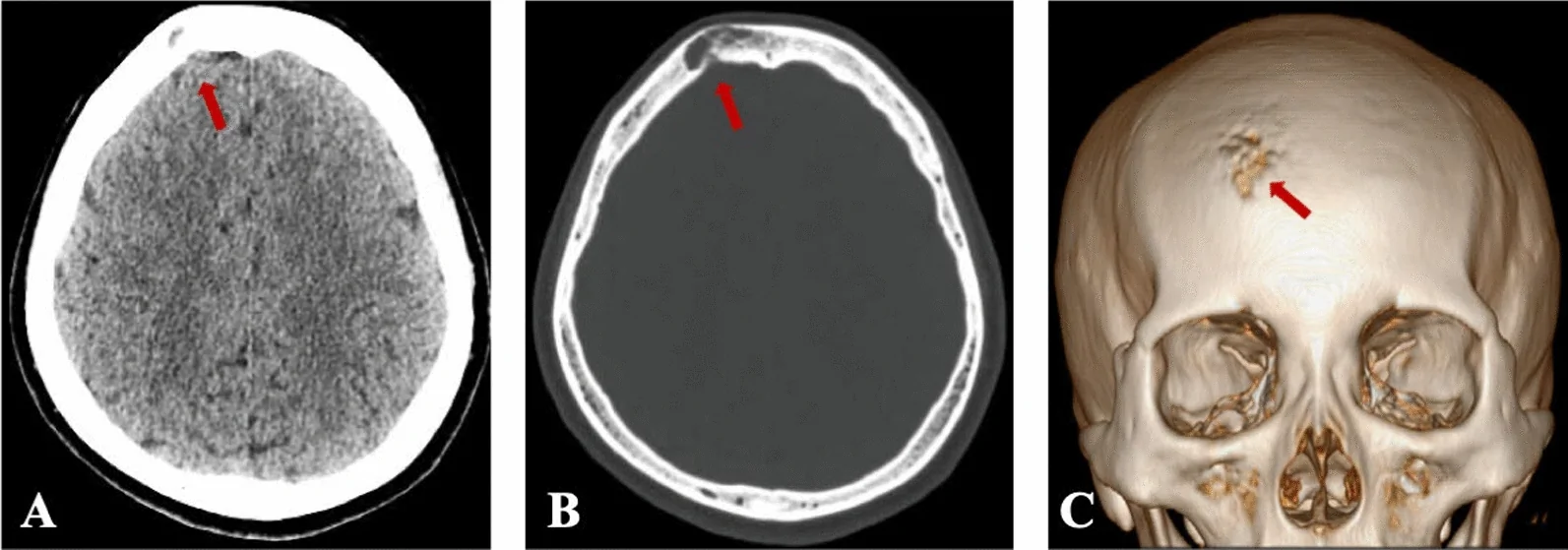
Preoperative head computed tomography (CT) images. **A**. CT in soft tissue window shows an oval hypodense lesion in the right frontal bone (arrow). **B**. CT in bone window clearly demonstrates the lesion as an area of osteolytic bone destruction with a well-defined border and internal fine osseous septations (arrow). **C**. A three-dimensional reconstruction image provides an intuitive view of the lesion extent and the arc-shaped indentation of the adjacent inner table (arrowhead)

Non-contrast head CT with 3D reconstruction revealed a well-defined, oval-shaped osteolytic defect in the right frontal bone (1.9 × 0.9 cm). The lesion exhibited internal fine septations and caused a characteristic focal depression of the adjacent inner table, suggesting an expansive but slow-growing process (Fig. 1).

**Fig. 2:**
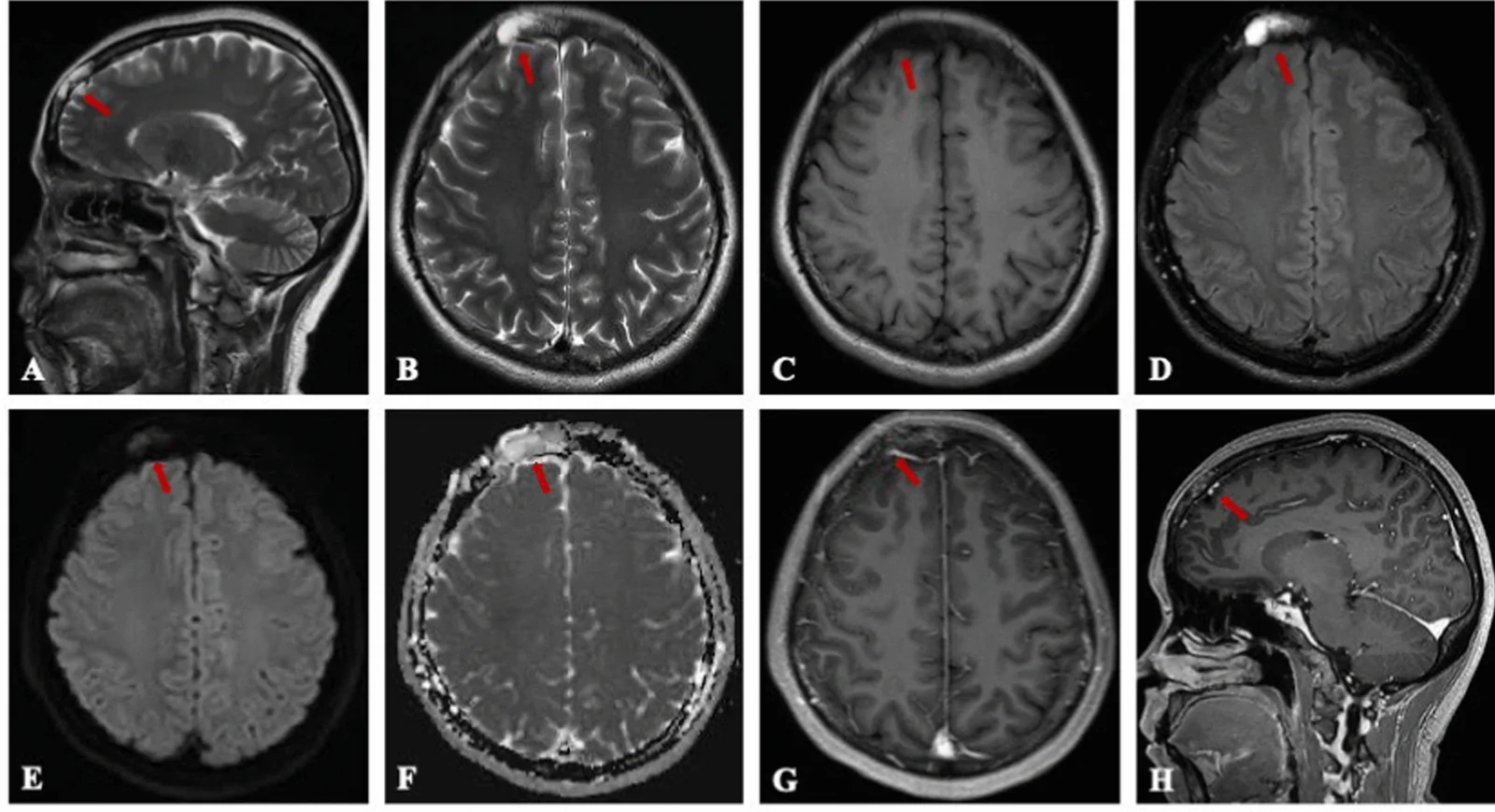
Preoperative head magnetic resonance imaging (MRI) images.** A**. Sagittal T2-weighted image (T2WI) shows a homogeneously hyperintense lesion (arrow). **B**. Axial T2WI demonstrates the lesion located within the diploë (arrow). **C**. Axial T1-weighted image (T1WI) shows a hypointense lesion (arrow). **D**. Axial T2-FLAIR image reveals a hyperintense lesion (arrow). **E**. Axial diffusion-weighted image (DWI) shows a hyperintense signal (arrow). **F**. Corresponding apparent diffusion coefficient (ADC) map shows no signal decrease (arrow). **G**. Axial post-contrast T1WI demonstrates heterogeneous enhancement of the lesion (arrow). **H**. Sagittal post-contrast T1WI further illustrates the enhancement pattern (arrow). No involvement of the adjacent brain parenchyma or dura mater is observed across all sequences

Brain MRI further characterized the lesion within the diploic space. The mass demonstrated marked hyperintensity on T2-weighted and T2-FLAIR sequences, and hypointensity on T1-weighted imaging. Notably, while the lesion showed high signal on Diffusion-Weighted Imaging (DWI), the corresponding Apparent Diffusion Coefficient (ADC) map showed no signal void. This mismatch is indicative of the "T2 shine-through" effect, reflecting low cellularity and high fluid content within the tumor. Post-contrast imaging showed heterogeneous enhancement (Fig. 2).

### Laboratory findings

Preoperative laboratory findings, including complete blood count, comprehensive metabolic panel, coagulation studies, and tumor markers, were unremarkable.

### Operative findings

**Fig. 3:**
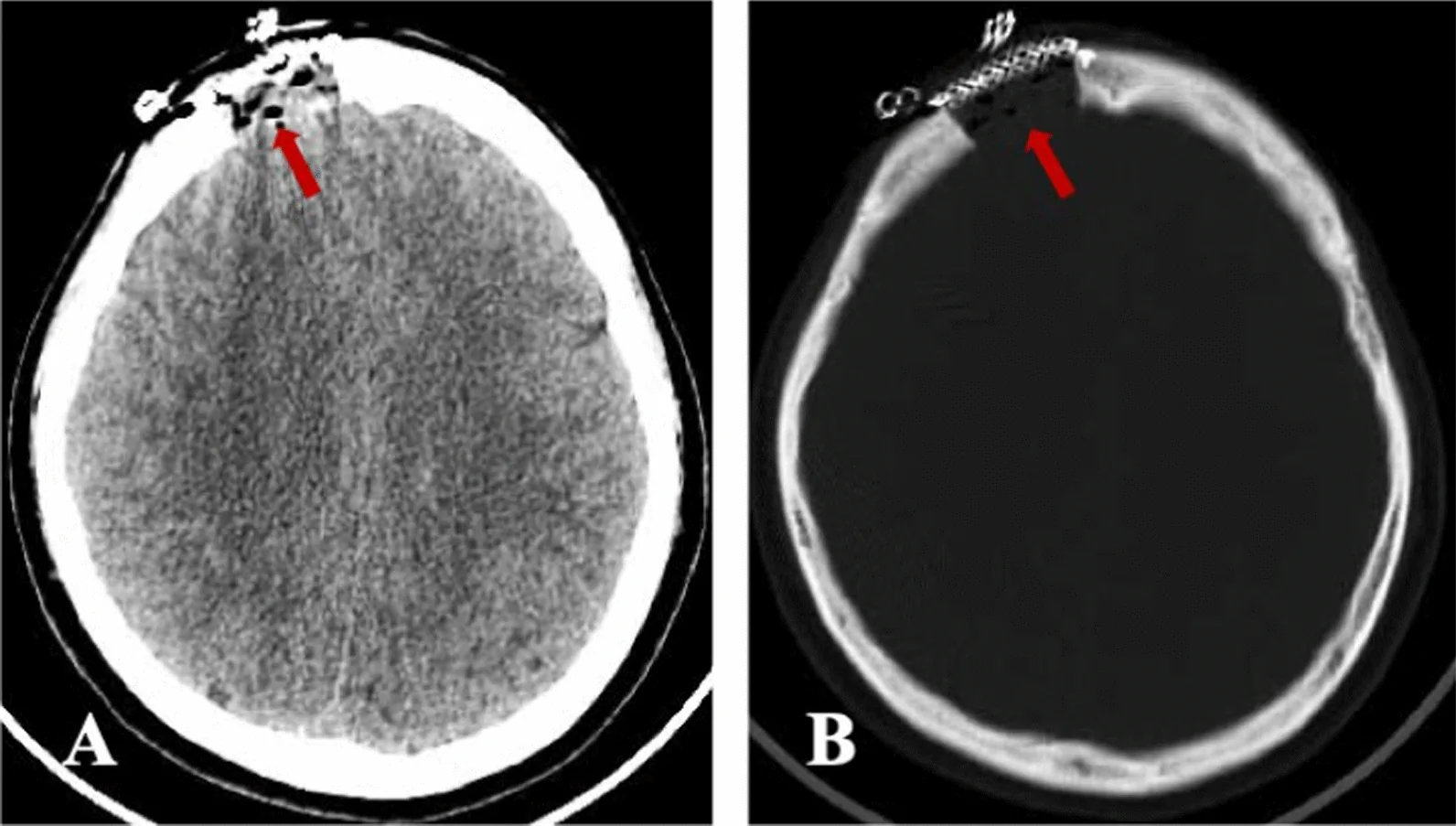
Postoperative head computed tomography (CT) images.** A**. CT in soft tissue window shows partial absence of the right frontal bone. The original bone defect is covered by a diamond-shaped titanium mesh. The underlying brain parenchyma appears well-maintained, with no definite hematoma or effusion in the operative region. **B**. CT in bone window clearly demonstrates the titanium mesh well-adapted to the skull defect and fixed with titanium screws. The position is satisfactory, without signs of loosening or displacement

Intraoperatively, a mass measuring approximately 2 cm was identified to the right of the midline in the frontal region. The lesion demonstrated expansive growth with an intact outer periosteum. Upon opening the lesion, friable, caseous material was encountered, and the underlying cranial bone showed expansive destruction. Complete excision of the mass was achieved. The dura underlying the lesion was found to be focally defective, and the defect was repaired using an artificial dural substitute. A diamond-shaped titanium mesh was then placed to cover the cranial defect and secured with titanium screws. Hemostasis was satisfactory, and the scalp was closed in layers. The procedure was uneventful. Postoperative imaging is shown in Fig. 3.

### Pathological findings

Microscopic examination of the resected specimen (Hematoxylin and Eosin stain, 200 × magnification) demonstrated meningothelial cell proliferation within the bony defect. The tumor exhibited a loose, microcystic architecture. The neoplastic cells contained clear cytoplasm and round to oval nuclei, with occasional intranuclear pseudoinclusions. Cell borders were indistinct. No cytological atypia, mitotic figures, or necrotic areas were identified.

**Fig. 4:**
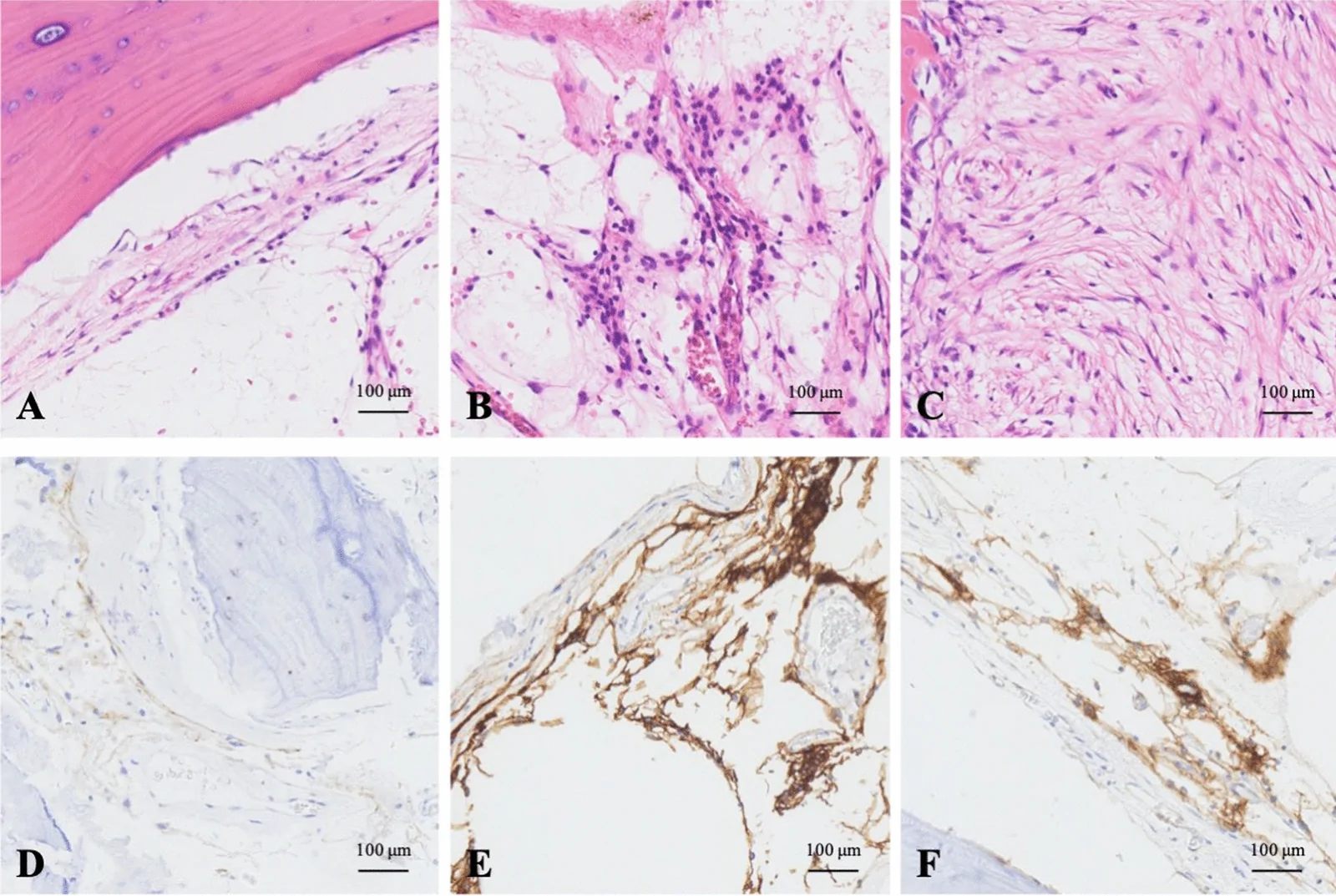
Histopathological and immunophenotypic characteristics of the tumor (microscopic view, × 200). **A**–**C** (H&E staining): The tumor cells are loosely arranged, forming prominent microcystic structures. The cells exhibit clear cytoplasm and round to oval nuclei, with occasional nuclear pseudoinclusions (arrows). Notable atypia or mitotic figures are absent. Adjacent bone tissue is visible at the periphery of the tumor. **D**–**F** (Immunohistochemical staining): The tumor cells show positive immunoreactivity for epithelial membrane antigen (EMA, **D**), somatostatin receptor 2 (SSTR2, **E**), and D2-40 (**F**). The Ki-67 proliferation index was low (< 2%, not shown). Scale bar: 100 μm (applicable to **A**–**F**)

Immunohistochemical analysis revealed positive staining for Epithelial Membrane Antigen (EMA), Somatostatin Receptor 2 (SSTR2), and D2-40 (Fig. 4).

### Pathological diagnosis

Right frontal skull lesion: Intraosseous Microcystic Meningioma (WHO Grade I). The morphological features and immunohistochemical profile are confirmatory.

### Follow-up outcomes

**Fig. 5:**
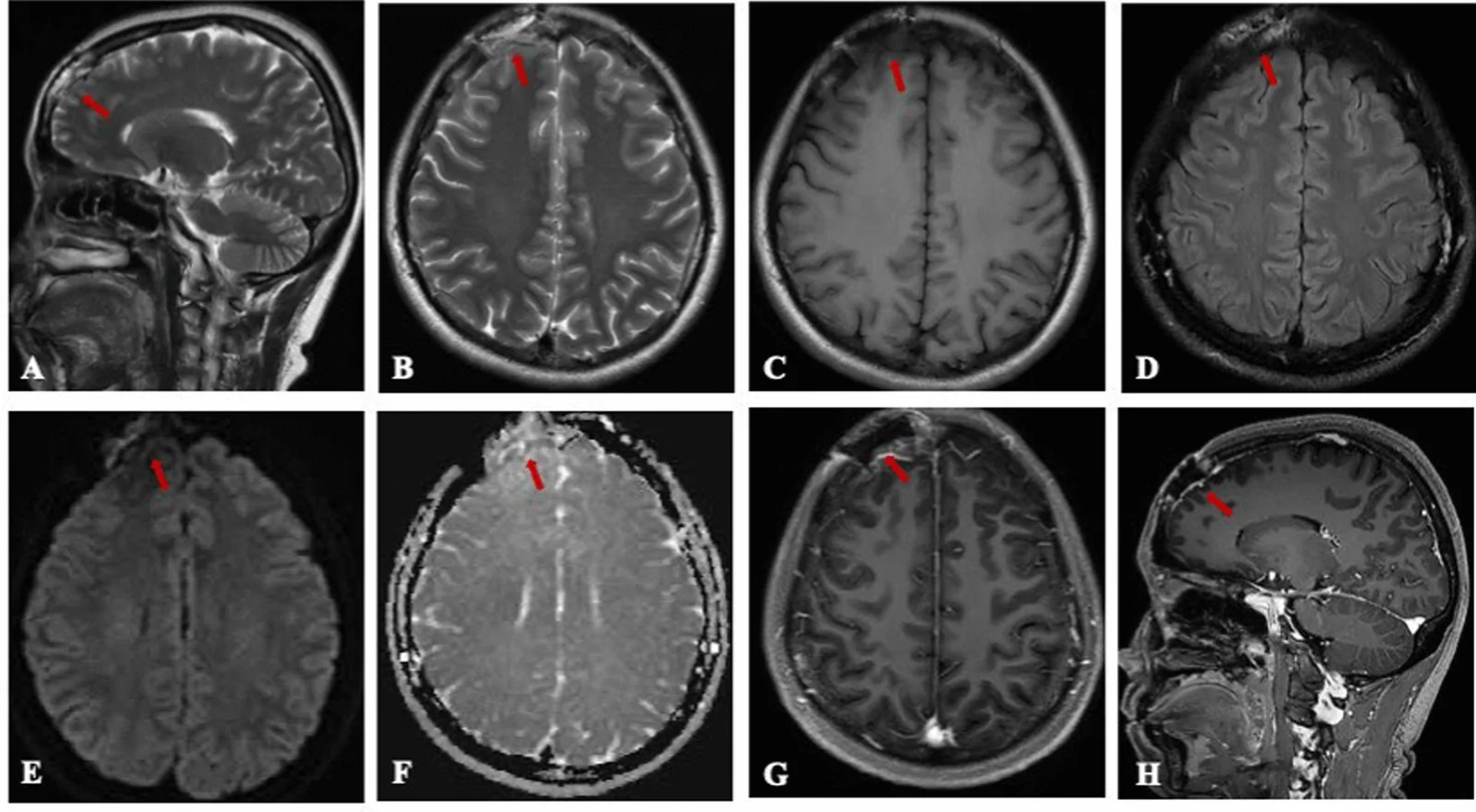
Postoperative follow-up brain MRI obtained 4 months after surgery.** A**. Sagittal T2-weighted image (T2WI) shows the titanium mesh as a signal-void structure (arrow) replacing the previous lesion site, with no abnormal hyperintensity. **B**. Axial T2WI demonstrates the titanium mesh well-adapted to the skull defect (arrow); no residual mass is seen within the diploic space. **C**. Axial T1-weighted image (T1WI) shows no hypointense lesion; the surgical site appears normal (arrow). **D**. Axial T2-FLAIR image reveals no hyperintense lesion; mild local postoperative changes are noted (arrow). **E**. Axial diffusion-weighted image (DWI) shows no abnormal hyperintense signal (arrow). **F**. Corresponding apparent diffusion coefficient (ADC) map shows no restricted diffusion (arrow). **G**. Axial post-contrast T1WI demonstrates no abnormal enhancement; physiological dural enhancement may be seen (arrow). **H**. Sagittal post-contrast T1WI further confirms the absence of enhancing tumor (arrow). No evidence of tumor recurrence or residual lesion is observed across all sequences. The adjacent brain parenchyma is unremarkable

At four-month postoperative follow-up (April 2, 2026), the patient reported complete headache resolution and had returned to all daily activities without neurological deficits. Physical examination revealed a well-healed surgical scar with no signs of infection or cerebrospinal fluid leakage. A follow-up brain MRI (plain and contrast) demonstrated stable positioning of the titanium mesh, no evidence of residual or recurrent tumor, and no new osteolytic lesions in the adjacent calvarium (Fig. 5). The patient had excellent adherence to the follow-up schedule. No adverse events or complications were recorded. Annual clinical and imaging surveillance is planned for five years.

## Discussion

Intraosseous microcystic meningioma is a rare tumor defined by its distinct anatomical origin and histopathological features, posing diagnostic challenges in clinical practice. Although sporadic cases have been reported, systematic reviews and comparative analyses remain limited. Herein, we present a new case and review previously reported cases (Table 2) [7, 11–14] to characterize the common clinical and imaging features of this entity while highlighting the unique aspects of the present case.

**Table 2:**
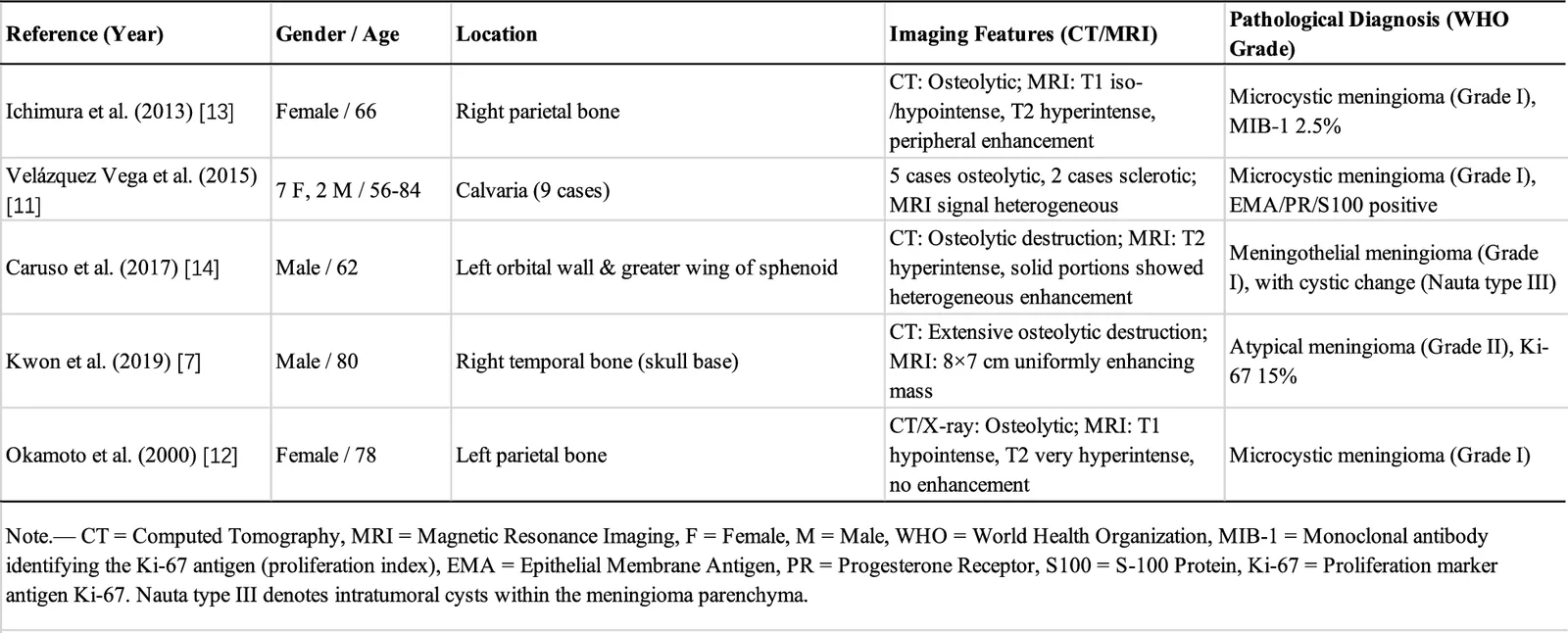
Characteristics of Literature-Reported Cases of Intraosseous Meningioma (including Microcystic/Cystic Subtypes)

As summarized from the literature (Table 2), intraosseous microcystic meningioma predominantly affects middle-aged to elderly women, with a median age of approximately 66 years, and typically manifests as a solitary osteolytic lesion. On MRI, these tumors frequently exhibit marked hyperintensity on T2WI and FLAIR sequences, correlating histopathologically with fluid accumulation within microcystic spaces. The present patient, aged 43 years, is notably younger, suggesting that the age spectrum of this disease may be broader than previously recognized.

Several imaging findings in this case merit further discussion. The CT features of inner table depression with internal septations suggest an indolent, pressure-remodeling process rather than aggressive osseous destruction—a pattern less emphasized in prior reports. More importantly, the DWI/ADC mismatch (T2 shine-through) observed here provides a potential preoperative clue: it indicates low cellular density and unrestricted water diffusion within microcysts, which may help differentiate this benign fluid-rich subtype from hypercellular malignancies such as metastases or myeloma that typically show true restricted diffusion. The heterogeneous enhancement pattern, though differing from the peripheral or mild enhancement described in some prior cases, likely reflects the uneven distribution of solid cellular components and microcystic spaces within the tumor.

Histopathologically, all reported cases, including the present one, correspond to WHO grade I meningioma.

Immunohistochemistry confirmed positive staining for EMA, SSTR2, D2-40, and PR, with occasional S-100 positivity and a low Ki-67 index (< 2%), consistent with a benign biological profile.

Notably, although microcystic meningioma is defined histologically, its characteristic MRI appearance—particularly prominent T2 hyperintensity—may offer valuable preoperative clues suggesting this subtype, especially in the intraosseous setting.

The differential diagnosis for intraosseous osteolytic lesions is broad (Table 3). Key considerations include hemangioma [15, 16], which typically exhibits a honeycomb or sunburst pattern on CT and flow voids on MRI, with vascular channels positive for CD31 and CD34; fibrous dysplasia [17, 18], characterized by ground-glass opacity on CT and woven bone with spindle cells on histology, often associated with GNAS mutation; multiple myeloma or metastatic carcinoma [19–21],presenting as multiple well-defined lytic lesions with CD138-positive plasma cells or cytokeratin-positive epithelial cells, respectively; and Langerhans cell histiocytosis [22–24], classically appearing as a punched-out lytic lesion with CD1a- and Langerin-positive Langerhans cells. Intraosseous microcystic meningioma is distinguished from these entities by its characteristic CT and MRI features and its immunoreactivity for EMA, PR, and SSTR2.

**Table 3:**
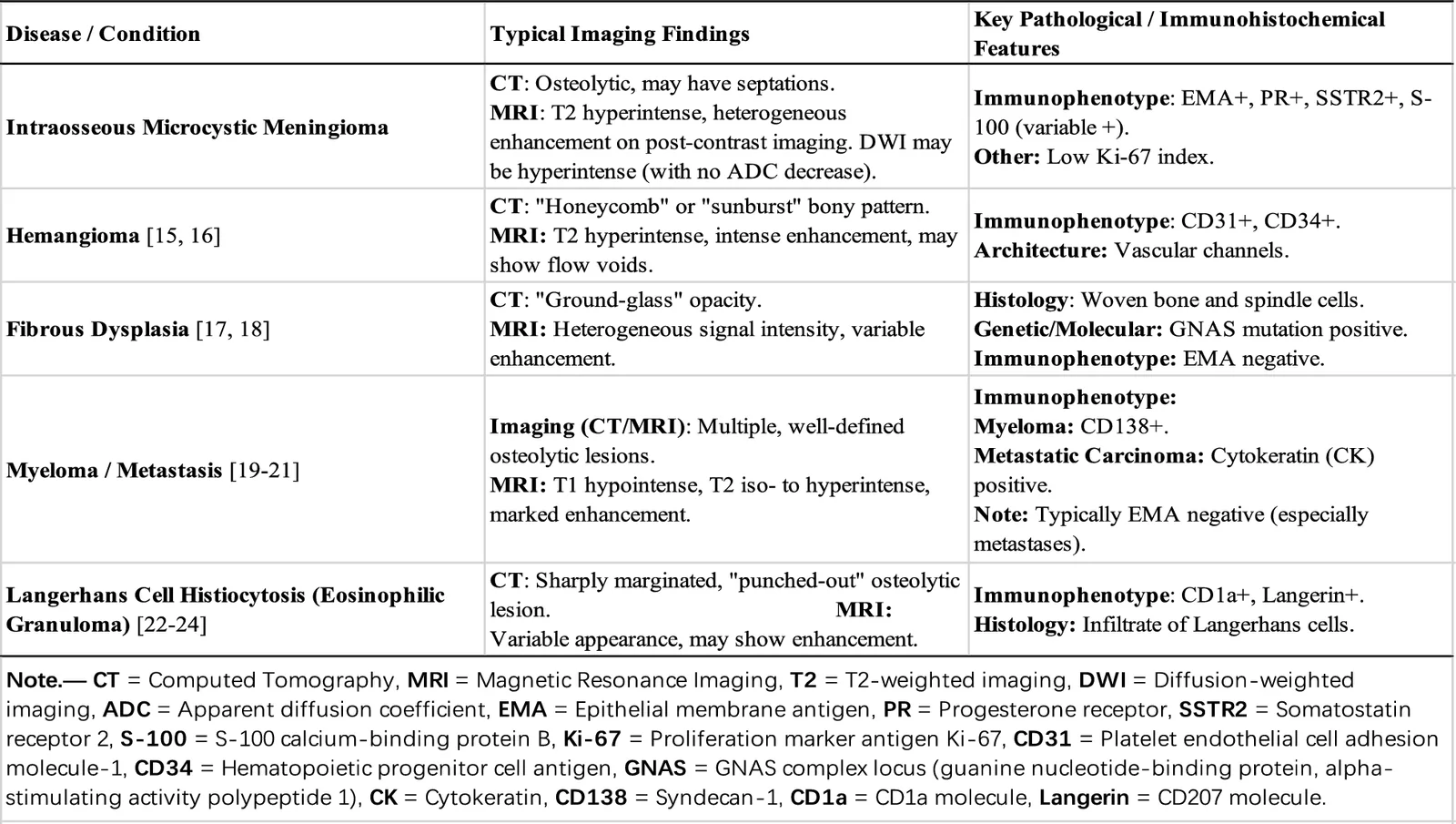
Key Diagnostic Features of Intraosseous Osteolytic Lesions

Complete surgical resection remains the treatment of choice and offers favorable outcomes. Reported cases treated with surgery alone have shown no recurrence, and our patient achieved gross total resection, supporting a good prognosis. For lesions in eloquent areas or where complete resection is not feasible, individualized consideration of adjuvant radiotherapy or close surveillance may be warranted.

This case contributes to the existing literature by documenting the disease in a younger patient and offering several insights. First, multiparametric MRI, particularly the combined interpretation of DWI and ADC values, can provide critical information for preoperative differential diagnosis. Second, intraosseous microcystic meningioma may be misdiagnosed as a cystic lesion or hemangioma due to its marked T2 hyperintensity; careful evaluation of enhancement patterns and diffusion characteristics is, therefore, essential. Future studies with prospective case collection and integrated clinical, imaging, and pathological data will help clarify the natural history of this disease and optimize diagnostic and therapeutic strategies.

### Strengths and limitations

This case report has several strengths. First, we provide detailed multiparametric MRI correlation with histopathology, particularly highlighting the T2 shine-through effect on DWI/ADC as a potential diagnostic marker for intraosseous microcystic meningioma. Second, we systematically compare our case with all previously reported cases in a summary table, offering a comprehensive literature review. Third, the patient’s relatively young age (43 years) extends the known age spectrum of this rare entity. Fourth, we present follow-up imaging at 4 months, confirming no early recurrence.

However, several limitations should be acknowledged. This is a single case report, which limits the generalizability of our findings. Long-term follow-up beyond 4 months is not yet available; therefore, the risk of late recurrence cannot be fully assessed. Additionally, we did not perform genetic or molecular profiling (e.g., NF2, AKT1, or TRAF7 mutations), which might have provided further insights into tumor pathogenesis. Finally, intraoperative frozen section was not obtained, and alternative advanced imaging (e.g., PET-CT) was not performed.

## Conclusion

In conclusion, intraosseous microcystic meningioma is a rare and easily misdiagnosed entity. Through this case report and literature review, we have systematically characterized its clinical, imaging, and pathological features, emphasizing the value of multimodal imaging in differential diagnosis. Although the tumor exhibits benign behavior and favorable surgical outcomes, heightened clinical awareness is necessary to ensure accurate diagnosis and appropriate management.

### Patient perspective

The patient shared: "I had a dull right-sided headache for six months. When I learned the CT scan showed a hole in my skull, I feared cancer. After surgery, my headache resolved immediately, recovery was smooth, and I went home within a week. I am grateful it was benign and needed no further treatment. Follow-up MRI confirmed everything is fine. I hope sharing my case helps others get the right diagnosis sooner."

## Data Availability

No datasets were generated or analysed during the current study.
